# From Manaus to Maputo: Toward a Public Health and Biodiversity Framework

**DOI:** 10.1007/s10393-014-0959-2

**Published:** 2014-07-04

**Authors:** Cristina Romanelli, Carlos Corvalan, H. David Cooper, Lucien Manga, Marina Maiero, Diarmid Campbell-Lendrum

**Affiliations:** 1 Secretariat of the Convention on Biological Diversity, United Nations Environment Programme, 413 St. Jacques, Suite 800, Montreal, H2Y1N9 Canada; 2 Pan American Health Organization and World Health Organization, Brasilia, Brazil; 3 World Health Organization Regional Office for Africa, Brazzaville, Congo; 4 World Health Organization, Geneva, Switzerland

**Keywords:** biodiversity, capacity-building, ecosystem services, post-2015 development agenda, health, sustainable development

## Abstract

The linkages between human health, biodiversity, ecosystems, and the life-supporting services that they provide are varied and complex. The traditional neglect of this nexus by policy-makers perpetuates threats posed to ecosystems with potentially critical impacts on global health. The Convention on Biological Diversity and the World Health Organization recently co-convened two regional workshops on these intricate but vital linkages. From discussions held with policy-makers and experts in the biodiversity and health sectors, spanning some 50 countries in Africa and the Americas, we derive a broad framework for the development of national and regional public health and biodiversity strategies relevant to strategic planning processes in the emerging post-2015 development context.

## Introduction

The structure and functioning of the world’s ecosystems has changed more rapidly in the second half of the twentieth century than in any other comparable period in human history (MA 2005). Some of these changes, such as increased food, energy, and water requirements, have been necessary to meet the needs of a rapidly growing population; however, they have also exacerbated ecosystem pressures and inequities (Myers et al. 2013), particularly among the world’s poorest, most vulnerable populations most immediately reliant on natural resources for food, shelter, medicines, spiritual and cultural fulfillment, and livelihoods (CBD 2010b; MA 2005).

While the understanding of how ecosystem alteration and degradation affect human health is incomplete, significant progress has been made toward understanding the scientific underpinnings at the biodiversity-health nexus, with a growing body of literature denoting that policy decisions affecting ecosystem management involve trade-offs (Rodríguez et al. 2006; Mace et al. 2012; Romanelli et al. 2014). A limited understanding of the benefits and challenges at the biodiversity–health interface and frequently corresponding failures to reflect these in policy decisions undermine our understanding of the full magnitude of health risks associated with biodiversity loss, ecosystem change, and the urgency required to address them (Jones et al. 2008; Pongsiri et al. 2009; Langlois et al. 2012; Stephens 2012; Myers et al. 2013; Keune et al. 2013).

Scientific progress toward understanding these linkages (see Box 1), and the socio-economic drivers by which they are influenced, has given momentum to holistic approaches such as EcoHealth and One Health (Webb et al. 2010; Parkes 2011; Romanelli et al. 2014) and to calls for enhanced collaboration between the World Health Organization (WHO) and the Convention on Biological Diversity (WHO 2012). In 2012, the WHO and the CBD Secretariat embarked on an unprecedented joint collaborative endeavor aimed at engaging health and biodiversity sectors worldwide, with particular emphasis on developing countries where concerted action is most urgently needed, in order to build capacity and promote action to jointly protect biodiversity and promote human health.

**Box 1:** Mutual Dependencies and challenges to biodiversity and human health

Human health and well-being depend on:
• The provision of adequate nutrition, clean water, medicines, and long-term food security provided by functioning ecosystems (WHO 2005; Hales and Corvalan 2006; Chivian and Bernstein 2008; Sala et al. 2009);
• Non-tangible benefits, known as cultural ecosystem services, such as spiritual values, recreational space, and cultural heritage (Rodríguez et al. 2006; MA 2005)
However, the linkages between biodiversity, ecosystem services and human health are multifaceted and complex (Rodríguez et al. 2006; Chivian and Bernstein 2008; Sala et al. 2009; Myers and Patz 2009; Stephens 2012; Myers et al. 2013). Challenges include:
• Concomitant pressures on the planet’s productive capacity and on the earth’s biological resources may undermine the ability of ecosystems to provide life-sustaining services (McMichael and Beaglehole 2000; CBD 2010a)
• Industrial food production, development of irrigation and energy supply systems have had net positive health benefits (Ersado 2005), but are often accompanied by unintended consequences including ground and surface water contamination, the release of harmful air pollutants, antimicrobial resistance, and health impacts related to the use of chemical pesticides (Myers et al. 2013; Horrigan et al. 2002; Mutero 2002)
• Positive feedback loops among climate change (Costello et al. 2009; McMichael et al. 2012), habitat alterations, land-use change (Foley et al. 2005), agricultural intensification (Tscharntke et al. 2012), invasive species (Mazza et al. 2014), urbanization (Bradley and Altizer 2007; Keune et al. 2013), poverty (Convention on Biological Diversity and Secretariat 2010b), and biodiversity loss (Herndon and Butler 2010; Díaz et al. 2006) can amplify a wide range of health threats, including malnutrition (McMichael et al. 2007; Fanzo et al. 2013), infectious diseases (Pongsiri et al. 2009; Jones et al. 2008), and non-communicable diseases (Alleyne et al. 2013; Beaglehole et al. 2011; Johns and Eyzaguirre 2006)

This collaboration is the result of significant international policy developments. In October 2010 and October 2012, the 10th and 11th Conference of the Parties (COP) to the CBD adopted decisions mandating a new era of closer collaboration between the Secretariat and the WHO (CBD COP Decision X/27; CBD COP Decision XI/VI) (Keune et al. 2013). Building on previous collaborative work with which the CBD and WHO have been involved (CBD 2008), including One Health, EcoHealth, and Co-Operation on Health and Biodiversity Initiative, among others, this strengthened partnership gave rise to the first two in a series of regional workshops led by these organizations in an effort to mainstream biodiversity and human health in biodiversity policies and strategies at the local, regional, and global levels. A significant advance of the Manaus and Maputo workshops is that it moves earlier international processes and recommendations to a regional level, highlighting and developing them for local concerns. As such, they can perhaps more directly facilitate cross-sectoral dialog and effective policy making at the national level.

Following a joint CBD-WHO workshop held at WHO headquarters in April 2012, the first of the regional workshops, for the Americas region, was held in Manaus, Brazil in 2012. It was followed by a regional workshop covering the whole of the Africa region, held in Maputo, Mozambique in 2013. The workshops aimed to foster collaborative work on the critical linkages between biodiversity, ecosystems, and public health, stimulate the development of effective public health and biodiversity strategies, and to enhance the implementation of related international commitments including the Strategic Plan for Biodiversity 2011–2020 (https://www.cbd.int/sp/) and its 20 Aichi Biodiversity Targets, which provides an agreed overarching framework for action on biodiversity and foundation for sustainable development for all stakeholders, including agencies across the United Nations system as a whole.

Moving from a global approach to regional workshops that draw on national experiences signifies real progress toward the goal of achieving greater integration between biodiversity and health policy. In this article, we draw on our discussions with the representatives from almost 50 countries in Africa and the Americas to designate a broad global framework for the development of robust regional and national strategies that reflect biodiversity–human health linkages. While no single approach can suffice for what are unique contexts, this broader framework can be used as a baseline for the development of more specific national and regional approaches. The discussion is particularly timely as the international community reviews its progress toward the fulfillment of Millennium Development Goals and proceeds toward a new global agreement on a post-2015 development agenda (UN 2012).

## The Workshops for the Americas and Africa

The workshops for the Americas (4–6 September 2012) and Africa (2–5 April 2013) regions brought together officials from the health sector and those responsible for the implementation of the CBD, as well as representatives from indigenous and local communities, international organizations, and experts in relevant fields.

The workshops were attended by a combined total of 108 participants from 49 countries, representing Ministries of Environment, Ministries of Health, as well as representatives from local and indigenous communities and from national, regional, and international organizations. Twenty-four countries from Latin America and the Caribbean were represented in the workshop for the Americas (www.cbd.int/en/health/americas) and representatives from 25 countries across the African continent were in attendance for the second regional workshop (www.cbd.int/en/health/africa). WHO and CBD focal points in the regions were invited to submit nominations, and a select number of organizations and experts with relevant regional expertise in the specific thematic issues identified were also present. Due to budgetary constraints, preference was given to Parties having submitted nominations from each the health and environment sectors. Where this was not possible, Parties from either the health or environment sector attended to ensure balanced regional representation. This is reflected in the resulting conclusions of the workshop embedded in the Framework proposed here.

Participants shared expertise and experience on a number of projects and programs at the health–biodiversity interface and in the implementation of National Biodiversity Strategies and Action Plans (NBSAPs), the principal instruments for implementing the Convention at the national level (https://www.cbd.int/nbsap/). The Convention requires countries to prepare an NBSAP (or equivalent instrument) and that this strategy be mainstreamed into the planning and activities of all sectors whose activities can have an impact (positive and negative) on biodiversity. To date, a total of 179 (92%) Parties have developed NBSAPs in line with Article 6 of the Convention. The 2008 Libreville Declaration on Health and Environment in Africa, aimed at addressing environmental impacts on environmental change on health in the region (WHO/UNEP 2008), and a number of local and national initiatives in the Americas were also discussed at length to derive a set of recommendations to promote further integration of biodiversity and health policies.

In addition to national expertise, the workshops gathered a range of science and policy experts to discuss a vast array of relevant issue areas focusing on water and food security, nutrition and non-communicable diseases, soil and air contamination, infectious and zoonotic diseases, traditional knowledge and medicines, cultural well-being, gender health, and natural resource management. Participants also had the opportunity to partake in field study visits to native rainforest and marine conservation areas, including the Bosque da Ciência in Manaus, Brazil, and the Marine Biological Station of Inhaca on Ihla dos Portugueses, Mozambique.

The sustainable management and use of biodiversity presents a broad range of opportunities for protecting both health and biodiversity, and for countries to develop related strategies and action plans. Examples of relevant issue areas and corresponding opportunities for the health sector addressed at the Manaus and Maputo workshops include:

*Food and nutrition* wildlife populations in terrestrial, marine, and freshwater systems are in decline as a result of habitat destruction, over-exploitation, pollution, invasive species, and other causes, presenting public health threats to human populations who depend on animal species for nutrition.

Improving quality, quantity, and supply of *water and other ecosystem services* can provide opportunities for the health sector to address the sources of disease, regulate disease, and integrate ecosystem management considerations into health policy while also promoting the protection and sustainable use of the ecosystems that supply these services.

*Disease control* land-use change and ecosystem disruption are widely recognized drivers of disease emergence. Targeted public health and biodiversity strategies provide opportunities to improve vector control, regulate and control the spread of emerging infectious diseases, zoonotic and other diseases as well as invasive alien species, and can contribute to ecosystem integrity, diversity, and the reconciliation of human development objectives.

*Traditional and modern medicines* derived from medicinal plants, animal species, and microbial organisms which provide opportunities for the health sector to recognize the contribution of genetic resources and traditional knowledge to medicine. They can also contribute to identifying and monitoring impacts of pollution from pharmaceutical sources (human, veterinary, and agricultural) on ecosystems, protecting genetic resources, and traditional knowledge, and ensuring the equitable sharing of benefits.

Benefits biodiversity provides to *physical, mental, and cultural well*-*being* include spiritual, recreational, and educational benefits as well as cultural enrichment. They also provide opportunities to integrate the “value of nature” into health policy, including mental health and non-communicable disease policies.

The urgent need for *climate change adaptation strategies* provides an opportunity for the health sector to help curtail the spread of pathogens, parasites, and diseases with potentially serious effects on human health that result from climate change and shifts in ecological conditions.

Participants in the regional workshops recognized that addressing biodiversity–health linkages can not only improve health and biodiversity outcomes but also contribute to livelihoods, poverty alleviation, disaster-risk reduction, and sustainable development more broadly, all of which are central to the burgeoning post-2015 development agenda. They called for the development of regional strategies and identified related elements essential to their success, based on national experiences. We draw upon these elements to sketch out a broad framework for the development of robust regional strategies.

## Toward a Framework for Public Health and Biodiversity Strategies

The United Nations Conference on Sustainable Development held in Rio de Janeiro in June 2012 (known as Rio +20), which resulted in the agreed outcome document *The Future We Want*, recognized the importance of and need to implement urgent global actions that promote both biodiversity conservation and public health. The section on Health and Population explicates the need to promote actions on social and environmental determinants of health, including for poor and vulnerable populations “to create inclusive, equitable, economically productive and healthy societies.” The document also emphasizes associations between biodiversity loss, ecosystem degradation, and human health, notably emphasizing “that these undermine global development, affecting food security and nutrition, the provision and access to water and the health of the rural poor and of people worldwide, including present and future generations” (UN 2012).

Health and biodiversity strategies should aim to ensure that the essential life-supporting services provided by ecosystems and vital biodiversity and health linkages are widely recognized, valued, and reflected in national public health and biodiversity strategies, and in the programs, plans, and strategies of other relevant sectors.

The implementation of such strategies should be a joint responsibility of ministries of health, environment, and other relevant ministries responsible for the implementation of environmental health programs and NBSAPs. The overall objective of the proposed framework is to guide the formulation of regional strategies and country-specific actions in the context of existing health and biodiversity commitments.

### Objectives

The framework should aim to reflect health–biodiversity linkages in relevant national policies and programs, specifically by:

- Promoting the health benefits provided by biodiversity for food security and nutrition, water supply, and other ecosystem services, traditional knowledge, cultures and food practices, the development of pharmacological sciences, pharmaceuticals and traditional medicines, mental health and poverty alleviation. In turn, this provides a rationale for the conservation and sustainable use of biodiversity as well as the fair and equitable sharing of benefits;
- Managing ecosystems to reduce the risks of infectious diseases, including zoonotic and vector-borne diseases, for example by avoiding ecosystem degradation, preventing invasive alien species, and limiting or controlling human-wildlife contact;
- Addressing drivers of environmental change (deforestation and other ecosystem loss and degradation and chemical pollution) that harm both biodiversity and human health, including direct health impacts and those mediated by biodiversity loss;
- Promoting lifestyles that might contribute jointly to positive health and biodiversity outcomes (e.g.,: protecting traditional foods and food cultures, promoting dietary diversity, etc.)
- Addressing the unintended negative impacts of health interventions on biodiversity (e.g.,: antibiotic resistance, contamination from pharmaceuticals), incorporating ecosystem concerns into public health policies, and addressing the unintended negative impacts of biodiversity interventions on health (e.g.,: effect of protected areas on access to food, medicinal plants, etc.).

### Priority Interventions Based on Workshop Conclusions

The framework is intended to encourage the implementation of a number of specific priority actions and interventions. Based on discussions held with country representatives and regional experts in the health and biodiversity communities in Africa and the Americas it was concluded that the international community and national governments should focus on a number of priority interventions, described in Box 2.

**Box 2:** Priority interventions based on workshop conclusions

(a) *Encourage the development of* *new and existing tools* such as environmental impact assessments, strategic environmental assessments, risk assessments, and health impact assessments that consider health-biodiversity linkages to manage future risks and safeguard ecosystem functioning while ensuring that social costs, including health impacts, associated with new measures and strategies do not outweight potential benefits
(b) *Strengthen core national capacities* that enable health systems to prepare for and effectively respond to public health threats resulting from ecosystem degradation and undertake cooperative actions toward capacity-building that promote the training of professionals in the health and biodiversity sectors, as well as indigenous and local communities
(c) *Promote research, development, and cooperation in traditional medicine* in compliance with national priorities and international legal instruments, including those concerning traditional knowledge and the rights of indigenous peoples, as appropriate
(d) Promote the *exchange of information, experiences, and best practices* to support the development of national and regional biodiversity and health strategies, and integrated tools of territorial planning
(e) *Disseminate and share lessons learned, knowledge, and national experiences* related to biodiversity–health linkages among countries and with international, national, and local partners to facilitate the development of tools aimed at integrating biodiversity in health strategies and reflecting public health considerations in biodiversity strategies
(f) Carry out *awareness raising activities* and *develop education programs* on the importance of health–biodiversity linkages at various levels, so as to enhance support for policies and their implementation
(g) *Promote further applied research on biodiversity*–*health linkages* to identify country-specific health risks, notably through disease organisms or ill-health triggers that result from ecosystem degradation and address local health adaptation needs and solutions. Research should also contribute to strengthening inter-country and regional research collaboration to address knowledge gaps and to incorporate social and cultural perspectives as well as traditional and religious values that serve to promote health and protect biodiversity
(h) Facilitate implementation of *integrated essential public health and biodiversity*-*related interventions* for the management of both short and long-term health risks resulting from biodiversity loss and unsustainable practices;
(i) Facilitate implementation of *integrated environment and health surveillance* to support timely and evidence-based decisions for the effective identification and management of short and long-term risks to human health posed by ecosystem degradation and biodiversity loss by forecasting and preventing increases in related ill-health and disease
(j) Strengthen and operationalize the *health components of disaster-risk reduction plans* to prevent casualties resulting from the health consequences of ecosystem degradation
(k) *Strengthen international and regional partnerships, joint work programs, and intersectoral collaboration* on biodiversity–health linkages

The proposed framework seeks to promote the deployment of an essential public health package to strengthen the biodiversity–health linkages in national policies, programs, plans, and strategies. The package consists of a set of interventions including comprehensive assessment of the risks to public health posed by ecosystem degradation, approaches to surveillance, and the delivery of preventive and curative interventions including preparedness for and response to the public health consequences of ecosystem degradation.

The implementation of interventions described in Box 2 is largely influenced by individual country institutional and financial capacities, and shaped by competing demands faced by health and environment agencies, with often limited resources. In that light, a pragmatic approach is needed, focusing first on those activities which require little initial investment and which will gradually develop partnerships and capacities to deliver more efficiently on the shared agendas of health and conservation actors. These are likely to include improved cross-sectoral collaboration mechanisms, the sharing of existing data and information, and the pooling of resources, where feasible. This would help to move beyond the confines of habitual institutional silos in which health and environmental policies are often developed, so interventions are no longer viewed as added burdens imposed by one sector on the other, but rather as important *opportunities* for collaboration toward improved health and conservation outcomes. It is also hoped that the workshops have generated momentum to extend efforts to other regions, encourage policy-makers to integrate joint biodiversity and health considerations into NBSAPs and national health strategies and eventually work toward an operative global framework within the context of the post-2015 development agenda being constructed by national Governments.

## Conclusion

Incorporating linkages at the biodiversity–health nexus in public health and conservation strategies will contribute not only to improved health and biodiversity outcomes but also to poverty alleviation, disaster-risk reduction, and sustainable development more broadly in line with the emerging post-2015 development agenda (Horwitz et al. 2012; Langlois et al. 2012). WHO and CBD, together with other partners, including the UN Convention to Combat Desertification and the UN Framework Convention on Climate Change, have made important advances in generating awareness and actions of the Rio Conventions by launching a discussion paper entitled *Our Planet, Our Health, Our Future* which was launched at the Rio +20 United Nations Conference on Sustainable Development (WHO 2012).

Based on existing programs and agreements, including global and regional strategies on climate change and health, specific agreements such the Libreville Declaration on Health and Environment in Africa, and the Interministerial Conferences in WHO Regions, the health community is becoming better placed to implement well-defined programs that jointly address biodiversity and health concerns. Similarly, the CBD has intensified collaboration with other conventions, agencies and partners, and key international commitments such as the Strategic Plan for Biodiversity 2011–2020 have been made under its auspices. Together, these developments provide significant opportunities to better understand and reflect biodiversity and health co-benefits in national, regional, and global policies and strategies (WHO 2012; Campbell et al. 2012). However, much more collaborative action—at all levels of governance—and applied research and policy implementation, are needed to further adapt and strengthen the framework outlined above. Its regional application also demands concerted efforts by international organizations, state governments, indigenous and local communities, conservation authorities and health sectors within countries.

Strong partnerships and information exchange networks are essential to transcending the “siloed” and frequently uncoordinated strategies of public health and biodiversity conservation sectors: they are foundational building blocks for mainstreaming biodiversity and public health concerns in national and regional plans and policies, and are central to strategic planning processes in the post-2015 development context. The directions charted in Manaus and Maputo can be instrumental to this endeavor, adapted to local contexts, and tailored to the needs and realities of different regions. Subjacent to this goal is the need to recognize that the achievement of public health goals, as expressed in the Millennium Development Goals and reflected in discussions on the burgeoning post-2015 development agenda, are dependent on our ability to maintain and sustain healthy ecosystems. The interdependence between ecosystem management and health outcomes must be reflected in local, regional, and global policies if we are to succeed.
